# The Frequency of Infant-Feeding Presentations at English Emergency Departments During the SARS-CoV-2 Pandemic: A Nation-Wide Electronic Health Records Study

**DOI:** 10.7759/cureus.27645

**Published:** 2022-08-03

**Authors:** Steven Wyatt, Patrick Aldridge, Samantha Ross, Sankara Narayanan, Luisa Zuccolo

**Affiliations:** 1 Health Policy, Strategy Unit, NHS Midlands and Lancashire Commissioning Support Unit, Birmingham, GBR; 2 Health Policy, Institute of Applied Health Research, University of Birmingham, Birmingham, GBR; 3 Emergency Medicine, Frimley Park Hospital, Frimley Health NHS Foundation Trust, Camberley, GBR; 4 General Practice, Dr Ross Practice, Shettleston Health Centre, Glasgow, GBR; 5 Neonatology, Watford General Hospital, West Hertfordshire Hospitals NHS Trust, Watford, GBR; 6 Population Health Sciences, Bristol Medical School, University of Bristol, Bristol, GBR; 7 Epidemiology and Public Health, Medical Research Council Integrative Epidemiology Unit, University of Bristol, Bristol, GBR

**Keywords:** neonatal jaundice, gastro-oesophageal reflux, sars-cov2, covid-19, breastfeeding, infant feeding, emergency department

## Abstract

Objectives: To examine the frequency and distribution of infant feeding-related presentations at emergency departments (EDs) before and during the SARS-CoV-2 pandemic.

Setting: Attendances at 48 major EDs in England in two 50-week periods before and during the COVID-19 pandemic: period 1, April 2, 2019 to March 10, 2020 and period 2, April 1, 2020 to March 10, 2021.

Methods: We estimated the change in frequency of ED presentations by age group and diagnosis before and after the start of the SARS-CoV-2 pandemic in England. We compared changes in the frequency of attendances of infant-feeding related presentations by infant age, sex, ethnicity, deprivation, rurality, arrival mode, arrival time, acuity, mother’s age, gravidity and mental health, birth length of stay, attendance duration, and disposal (i.e., admission or discharge).

Results: While total ED attendances fell by 16.7% (95% CI -16.8% to -16.6%), infant attendances increased for feeding problems (+7.5% 95% CI 2.3% to 13.0%), neonatal jaundice (+12.8%, 95% CI 3.3% to 23.3%) and gastro-esophageal reflux (+9.7%, 95% CI 4.4% to 15.2%). These increases were more pronounced amongst first babies (+22.4%, 95% CI 13.1% to 32.5%), and where the stay in hospital after birth was brief (0-1 days, +20.1%, 95% CI 14.8% to 25.7%). Our analysis suggests that many of these attendances were of low acuity.

Conclusions: While ED attendances reduced dramatically and systematically with the COVID-19 pandemic, presentations for infant feeding issues increased, implying growth in the unmet needs of new mothers and infants.

## Introduction

The severe acute respiratory syndrome coronavirus 2 (SARS-CoV-2) first emerged in 2019 and causes coronavirus disease 2019 (COVID-19). The World Health Organization declared it a Public Health Emergency in January 2020 and a pandemic in March 2020 [1]. In efforts to limit transmission, governments implemented a range of non-pharmaceutical interventions including physical distancing measures, such as closures of schools, retail businesses, and restaurants as well as restrictions on individual movements and social interactions [2,3].

Collectively these non-pharmaceutical interventions, commonly known as “lockdown” measures, led to substantial changes to healthcare provision. In England, healthcare policies and guidance sought to free-up capacity to manage the anticipated rise in COVID-19 cases. Many hospital and community health care services were temporarily suspended, curtailed, or redesigned [4]. Maternity and newborn services were no exception.

While several studies demonstrated substantial reductions in emergency department (ED) attendances for many months after the COVID-19 outbreak, there were reports that attendances of infants had fallen more modestly, or even increased, with a particular emphasis on presentations relating to infant feeding [5-7].

Problems with infant feeding can result in acute presentations such as neonatal jaundice and gastro-esophageal reflux disease (GORD). Up to 60% of healthy term newborns are reported to be visibly jaundiced in the first week of life [8]. Early identification of feeding difficulties and referral for community-based feeding support are key preventative factors for severe jaundice. Gastro-esophageal reflux (GOR) is an effortless involuntary regurgitation of gastric contents into the esophagus that does not require treatment and usually resolves by the age of one year. In contrast, GORD is used to describe instances where an infant exhibits pain, distress, or feed aversion with faltering weight. Estimating the prevalence of GORD in infants is difficult due to variation in both presentation and diagnosis. However, an Australian study estimated this at 0.5% in a representative primary care population [9]. We examine the frequency and distribution of infant feeding-related presentations, including neonatal jaundice and GORD at EDs in England following the SARS-CoV-2 pandemic.

## Materials and methods

Analysis, setting, and population

Our analysis was centered on attendances at consultant-led EDs at 48 NHS Trusts in England, between April 2, 2019 and July 7, 2021. We calculated weekly counts of all ED attendances, attendances of infants (< 1 year), and infants with feeding-related problems.

We compared counts of ED attendances over two comparable, 49 weeks, time periods, by age group. Period 1, ran from April 2, 2019 to March 10, 2020 and period 2, from April 1, 2020 to March 10, 2021. For infants (<1 year) we compared counts of ED attendances over the two periods by diagnosis. For ED attendances of infants (<1 year) with feeding-related problems, we compared counts of ED attendances over the two periods by several patient characteristics (age group, sex, ethnicity, the level of deprivation, and rurality of the patient’s area of residence, mother’s age, gravidity, mental health status and birth episode length of stay) and attendance characteristics (arrival mode, acuity, the day of week and time of day of attendance, attendance duration and disposal).

Diagnosis recording in EDs is not complete. Given our focus was on infants with feeding presentations, we limited our analysis to 48 of the 125 NHS Trusts with consultant-led EDs that supplied diagnoses codes for at least 75% of ED attendances each week during the study period.

Annual data on births, breastfeeding initiation and breastfeeding prevalence at six to eight weeks were collated and summarized to provide contextual information, and aid interpretation of the results. Data on births provides information about changes in the size of the population at risk, while breastfeeding trends offer insight into changes in a key risk factor.

Confidence intervals for the ratio of two counts were derived using Monte-Carlo simulation with 10,000 replications and the standard error of the underlying counts, assuming these followed a Poisson distribution. All analyses were undertaken using R v 4.0.3 [10].

Variables and data sources

Anonymized extracts of the Emergency Care (ECDS) and Admitted Patient Care (APC spells) datasets were obtained from the National Commissioning Data Repository (NCDR) administered by NHS England [11,12]. These datasets are derived from real-time information systems maintained by clinical and administrative staff in NHS Trusts and are used to support service improvement, planning and research activities. Access to these datasets for individuals not employed by NHS England is controlled by NHS Digital. The extracts contained demographic, administrative, and clinical information about all attendances at the selected ED departments in England between April 2019 and July 2021, any subsequent emergency hospital admissions for these patients, and in the case of infants, the birth spell of the mother and infant.

Attendances were grouped by several demographic, socio-economic, clinical presentation, outcome and birth variables: sex (male, female, other, not known), age group (<1 week, 2-4 weeks, 2-3 months, 4-6 months, 7-9 months, 9-12 months, 1-4 years, 5-19 years, 20-44 years, 45-69 years, 70+ years), ethnicity (White British, White Irish, White other, Indian, Pakistani, Bangladeshi, Asian other, Black Caribbean, Black African, Black other, Mixed - White and Black Caribbean, Mixed - White and Black African, Mixed - White and Asian, Mixed - other, Chinese, other ethnic group, not known, not stated), deprivation (quintiles), urbanicity (urban, rural), mother’s age at birth (<20 years, 20-29 years, 30-39 years, 40+ years), gravidity (0, 1+ previous pregnancies), mental health comorbidities, birth length of stay (0-1 days, 2-4 days, 5+ days), arrival mode (by ambulance, self-conveyed), acuity (immediate, very urgent, urgent, standard, not urgent), primary diagnosis (Snomed CT), arrival day of week (weekday, weekend), time of day (8am to 8pm, 8pm to 8am) and disposal (admitted, not admitted).

Deprivation was defined using the English Indices of Deprivation 2015 [13]. This area-based measure was assigned based on the Lower Super Output Area of residence of the patient. Urbanicity was defined using the Rural and Urban Classification 2004 [14]. Patient diagnoses were taken from Snomed CT codes recorded in ECDS [15].

Infant feeding related ED presentations were defined as those aged <1 year with one of the following primary diagnoses, Snomed CT codes: 72552008 (feeding problem in new-born), 235595009 (GORD), and 387712008 (neonatal jaundice). Mental health comorbidities of the mother were derived diagnoses (ICD1-10 Chapter F, excluding F17.0-F17.9) in the intrapartum hospital spell. Summary data on births was obtained from the Office of National Statistics [16]. Summary data on breastfeeding initiation and breastfeeding prevalence at six to eight weeks were obtained from NHS England, Public Health England, NHS Digital, and the Nuffield Trust [17-20].

## Results

Frequency and distribution of attendances at baseline

In the 50-week period prior to the first national lockdown, there were 5.9 million attendances at the EDs in the study. Of these 155.8 thousand (2.6%) were for infants aged less than one year (Table 1).

**Table 1:** Attendances by age group during 50-week period prior to lockdown (April 2, 2019 to March 10, 2020) at 48 major EDs in England.

Age group	Attendances	%
all	5,907,139	100.0%
Under 1 year	155,848	2.6%
1-4 years	340,043	5.8%
5-19 years	838,486	14.2%
20-44 years	1,854,421	31.4%
45-69 years	1,432,088	24.2%
70+ years	1,286,020	21.8%
Not given	233	0.0%

Of these infant presentations and where data was available, 10.6% were for those aged under four weeks, 56.0% were male, 28.8% were living in the most deprived quintile of areas in England, and 87.6% were living in urban areas. Tracing back to the hospital birth spell, we find that 61.8% of infant ED attendances were for those whose mother was aged under 30 years at birth, 33.0% were for mothers with no previous pregnancies, 17.1% had a recorded comorbid mental health problem, and 44.3% spent more than one night in the hospital during the intrapartum spell (Table 2).

**Table 2:** Characteristics of infant (<1 year) attendances during 50-week period prior to lockdown (April 2, 2019 to March 10, 2020) at 48 major EDs in England.

Group	Subgroup	Attendances	%	% (where data available)
All	All	155,848	100.0%	100.0%
Age group	0-1 weeks	182	0.1%	0.1%
	2-4 weeks	16,389	10.5%	10.5%
	2-3 months	31,020	19.9%	19.9%
	4-6 months	31,819	20.4%	20.4%
	7-9 months	36,198	23.2%	23.2%
	10-12 months	40,240	25.8%	25.8%
Sex	Male	86,027	55.2%	56.0%
	Female	67,498	43.3%	44.0%
	Not given/not known	2,323	1.5%	-
Ethnicity	White - British	98,328	63.1%	68.9%
	White - Irish	502	0.3%	0.4%
	White - Any other White background	13,668	8.8%	9.6%
	Mixed - White and Black Caribbean	1,926	1.2%	1.3%
	Mixed - White and Black African	1,062	0.7%	0.7%
	Mixed - White and Asian	1,751	1.1%	1.2%
	Mixed - Any other mixed background	3,831	2.5%	2.7%
	Asian or Asian British - Indian	2,557	1.6%	1.8%
	Asian or Asian British - Pakistani	5,377	3.5%	3.8%
	Asian or Asian British - Bangladeshi	1,486	1.0%	1.0%
	Asian or Asian British - Any other Asian background	2,847	1.8%	2.0%
	Black or Black British - Caribbean	812	0.5%	0.6%
	Black or Black British - African	3,846	2.5%	2.7%
	Black or Black British - Any other Black background	997	0.6%	0.7%
	Other Ethnic Groups - Chinese	493	0.3%	0.3%
	Other Ethnic Groups - Any other ethnic group	3,268	2.1%	2.3%
	Not given/not known	13,097	8.4%	-
Deprivation quintile	Quintile 1 - most deprived	44,516	28.6%	28.8%
	Quintile 2	32,487	20.8%	21.0%
	Quintile 3	28,683	18.4%	18.5%
	Quintile 4	25,877	16.6%	16.7%
	Quintile 5 - least deprived	23,236	14.9%	15.0%
	Not given/not known	1,049	0.7%	-
Urban/rural	Urban	135,719	87.1%	87.6%
	Rural	19,299	12.4%	12.4%
	Not given/not known	830	A0.5%	
Age group of mother	Under 20 years	8,034	5.2%	8.7%
	20-29 years	49,140	31.5%	53.2%
	30-39 years	33,615	21.6%	36.4%
	40+ years	1,661	1.1%	1.8%
	Not given/not known	63,398	40.7%	-
Previous pregnancies	None	26,183	16.8%	33.0%
	1 or more	53,215	34.1%	67.0%
	Not given/not known	76,450	49.1%	-
Intrapartum length of stay	0-1 night	81,300	52.2%	55.7%
	2-4 nights	46,955	30.1%	32.2%
	5+ nights	17,761	11.4%	12.2%
	Not given/not known	9,832	6.3%	-
Maternal mental health	Comorbid MH	15,801	10.1%	17.1%
	No comorbid MH	76,649	49.2%	82.9%
	Not given/not known	63,398	40.7%	-

Considering the attendance characteristics of infant ED attendances during this period, we found, where data was available that 21.4% had been conveyed by ambulance, 15.2% were regarded as requiring immediate or very urgent attention, 30.2% arrived on a weekend and 36.9% between 8 pm and 8 am, 5.0% related to an infant feeding issue (feeding problem in newborn, neonatal jaundice or GOR), 36.0% spent less than two hours in ED and 26.3% were admitted on disposal (Table 3).

**Table 3:** Characteristics of infant (<1 year) attendances during 50-week period prior to lockdown (April 2, 2019 to March 10, 2020) at 48 major EDs in England.

Group	Subgroup	Attendances	%	% (where data available)
All	All	155,848	100.0%	100.0%
Arrival mode	Ambulance	33,060	21.2%	21.4%
	Walk-in	121,069	77.7%	78.6%
	Not given/not known	1,719	1.1%	-
Acuity	Immediate	1,969	1.3%	1.4%
	Very urgent	20,107	12.9%	13.8%
	Urgent	44,247	28.4%	30.4%
	Standard	73,204	47.0%	50.3%
	Non-urgent	5,944	3.8%	4.1%
	Not given/not known	10,377	6.7%	-
Part of week	Weekday	108,790	69.8%	69.8%
	Weekend	47,058	30.2%	30.2%
Part of day	Day (8am-8pm)	98,356	63.1%	63.1%
	Night (8pm-8am)	57,492	36.9%	36.9%
Diagnoses	Feeding problem in newborn	3,048	2.0%	2.2%
	Neonatal jaundice	919	0.6%	0.6%
	Gastro-esophageal reflux	3,044	2.0%	2.2%
	Upper respiratory tract infection	18,883	12.1%	13.3%
	Infectious gastroenteritis	6,411	4.1%	4.5%
	Traumatic brain injury - no loss of consciousness	5,274	3.4%	3.7%
	Croup	3,988	2.6%	2.8%
	Bronchiolitis	21,330	13.7%	15.1%
	Other diagnosis	78,608	50.4%	42.1%
	No abnormality detected	19,028	12.2%	13.4%
	None recorded	14,343	9.2%	-
ED duration	0-1hr	20,513	13.2%	13.2%
	1-2hrs	35,625	22.9%	22.9%
	2-3hrs	39,554	25.4%	25.4%
	3-4hrs	40,857	26.2%	26.2%
	4+hrs	19,248	12.4%	12.4%
	Not given/not known	51	0.0%	-
ED disposal	Admitted	40,920	26.3%	26.3%
	Not admitted	114,928	73.7%	73.7%

The age and sex distribution of attendances at the major EDs selected for this study was similar to the profile of attendances at all major EDs in England (Table 4).

**Table 4:** Age and sex profile of ED attendances during 50-week period prior to lockdown in study sites and all major EDs in England.

Subgroup	Study site EDs	All major EDs
Under 1 year	2.6%	3.0%
1-4 years	5.8%	6.1%
5-19 years	14.2%	14.3%
20-44 years	31.4%	31.9%
45-69 years	24.2%	23.9%
70+ years	21.8%	20.7%
Age not given	0.0%	0.0%
Male	48.5%	48.9%
Female	50.1%	50.6%
Sex not given/not known	1.4%	0.5%

Change in frequency of attendances

**Figure 1:**
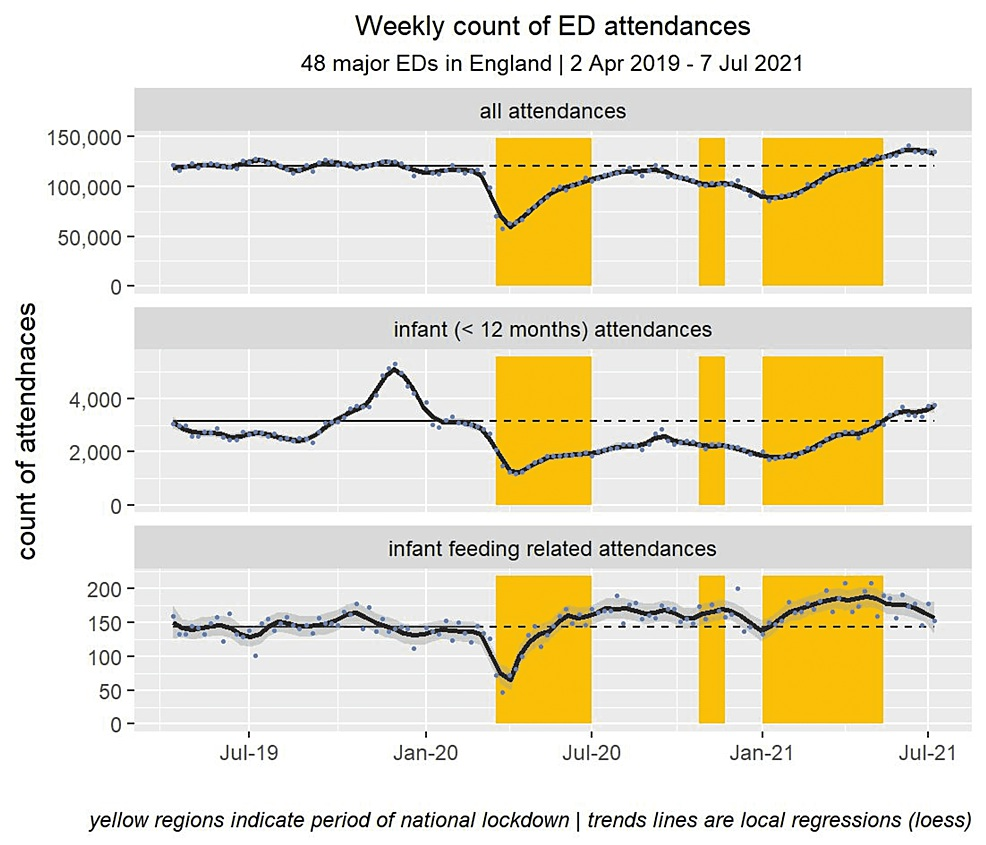
Weekly count of ED attendances at 48 major EDs in England (April 2, 2019 to July 7, 2021).

ED attendances of all types fell sharply immediately after the introduction of the first national lockdown in England on March 23, 2020 (Figure 1). Weekly attendance counts remained below the pre-pandemic average for more than 12 months. Attendances for infant feeding-related issues, however, rose beyond the pre-pandemic levels, mid-way through the first national lockdown and remained above this level for most of the subsequent 12-month period, dipping only marginally below this level in the weeks leading up to the new year of 2021.

**Figure 2:**
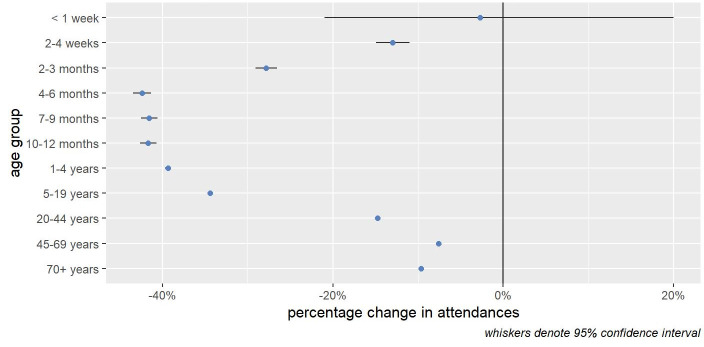
Change in ED attendances by age group.

**Figure 3:**
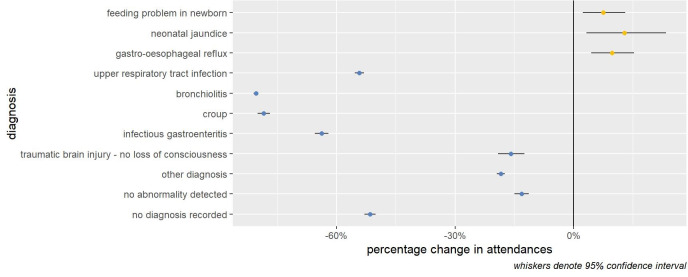
Change in infant ED attendances by selected diagnoses: 48 major EDs in England (April 2, 2019 to March 10, 2020) vs (April 1, 2020 to March 10, 2021).

Comparing two 50-week periods before and during the pandemic (April 2, 2019 to March 10, 2020 and April 1, 2020 to March 10, 2021), we note that total ED attendance of all ages fell by 16.7% (95% CI -16.8% to -16.6%). Figures 2, 3 illustrate that these changes varied considerably by age group with smaller reductions seen in the very young (<1 month) and those aged over 45 years.

Focusing on attendances for infants (<1 year), we find that while attendances for most diagnoses reduced in the 50 weeks after the start of the pandemic, attendances increased for feeding problems in newborns (+7.5% 95% CI 2.3 to 13.0%), neonatal jaundice (+12.8%, 95% CI 3.3% to 23.3%) and GOR (+9.7%, 95% CI 4.4% to 15.2%) (Table 2, Figure 2). Taken together, presentations for these three infant feeding-related issues increased by 8.9% (95% CI 5.4% to 12.5%). For infants (<1 year) we find that feeding-related attendances (feeding in newborn, neonatal jaundice, and GORD) made up 4.5% (n = 7,059) of ED attendances <1 years old in the 50-week period pre-pandemic and which increased to 7.7% (n = 7,705) in the 50-week period after the start of the pandemic (Tables 5, 6).

**Table 5:** Change in ED attendances by age group between two 50-week periods prior to and during lockdown at 48 major EDs in England.

Age group	Attendances period 1	Attendances period 2	% change	95% CI
< 1 week	182	177	-2.7%	[-21.0% to 20.0%]
2-4 weeks	16,389	14,258	-13.0%	[-14.9% to -11.0%]
2-3 months	31,020	22,391	-27.8%	[-29.0% to -26.6%]
4-6 months	31,819	18,329	-42.4%	[-43.4% to -41.3%]
7-9 months	36,198	21,159	-41.5%	[-42.5% to -40.5%]
10-12 months	40,240	23,473	-41.7%	[-42.6% to -40.7%]
1-4 years	340,043	206,224	-39.4%	[-39.7% to -39.0%]
5-19 years	838,486	549,842	-34.4%	[-34.6% to -34.2%]
20-44 years	1,854,421	1,580,843	-14.8%	[-14.9% to -14.6%]
45-69 years	1,432,088	1,323,741	-7.6%	[-7.8% to -7.3%]
70+ years	1,286,020	1,162,101	-9.6%	[-9.9% to -9.4%]

**Table 6:** Change in infant (<1 year) ED attendances by diagnosis between two 50-week periods prior to and during lockdown at 48 major EDs in England. * no loss of consciousness

Selected diagnoses	Attendances period 1	Attendances period 2	% change	95% CI
feeding problem in newborn	3,048	3,277	7.5%	[2.3% to 13.0%]
neonatal jaundice	930	1,049	12.8%	[3.3% to 23.3%]
gastro-oesophageal reflux	3,081	3,379	9.7%	[4.4% to 15.2%]
upper respiratory tract inf.	18,970	8,687	-54.2%	[-55.4% to -53.0%]
infectious gastroenteritis	6,618	2,398	-63.8%	[-65.4% to -62.0%]
traumatic brain injury*	5,322	4,478	-15.9%	[-19.1% to -12.4%]
croup	4,046	873	-78.4%	[-80.0% to -76.8%]
bronchiolitis	21,718	4,268	-80.3%	[-81.0% to -79.7%]
no abnormality detected	19,129	16,606	-13.2%	[-15.0% to -11.4%]
no diagnosis recorded	14,343	6,949	-51.6%	[-52.9% to -50.1%]
other diagnosis	58,643	47,823	-18.5%	[-19.4% to -17.5%]

Subgroup analysis

**Figure 4:**
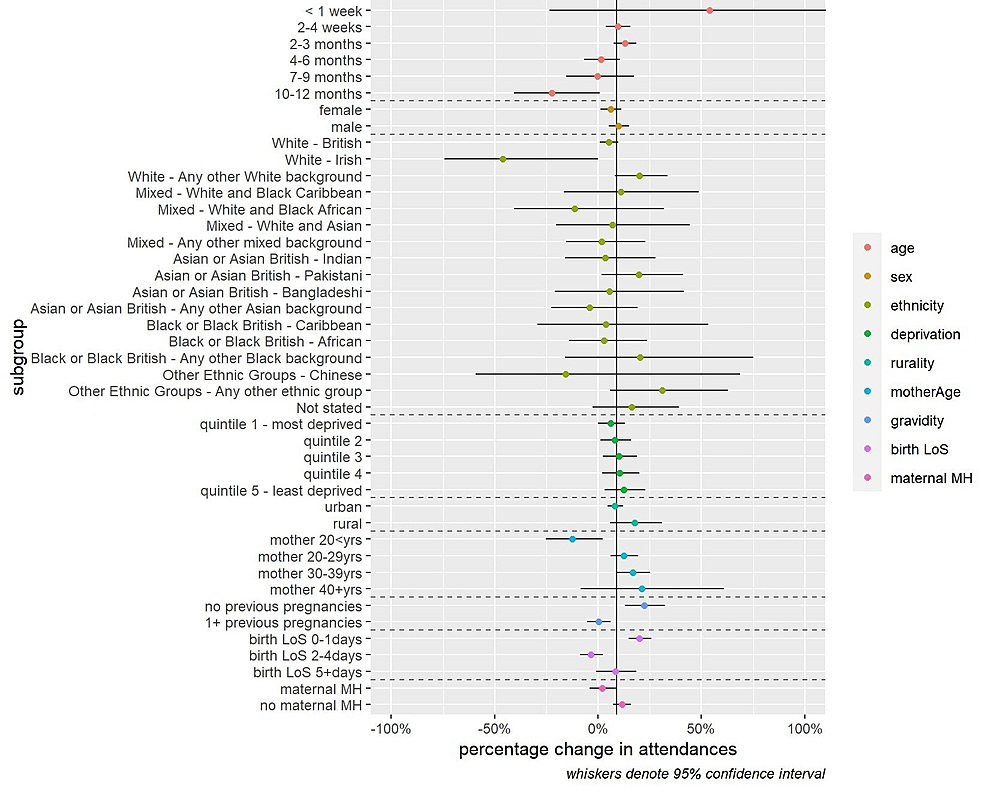
Change in ED attendances for infants with feeding-related problems by patient characteristics: 48 major EDs in England (April 2, 2019 to March 10, 2020) vs (April 1, 2020 to March 10, 2021).

These increases in infant feeding-related presentations were broadly consistent across many patient characteristics. We note however significantly lower increases (or modest reductions) amongst infants aged 10-12 months (-22.5%, 95% CI -40.7% to 0.9%), of White Irish ethnicity (-46.1%, 95% CI -74.3% to 0.0%), born to mothers aged under 20 years (-12.6%, 95% CI -25.3% to 2.3%), to mothers who had had 1 or more previous pregnancies (+0.2%, 95% CI -5.4% to 6.1%) or whose intrapartum spell had lasted two to four days (-3.4%, 95% CI -8.8% to 2.4%). We noted significantly larger increases in infant feeding presentations for infants of mothers who had no previous pregnancies (+22.4%, 95% CI 13.1% to 32.5%) or whose intrapartum spell lasted zero to one day (20.1%, 95% CI 14.8% to 25.7%). We observed no significant variation in the increase in infant feeding presentations by sex, deprivation, rurality, or maternal mental health status at birth (Figure 4).

**Figure 5:**
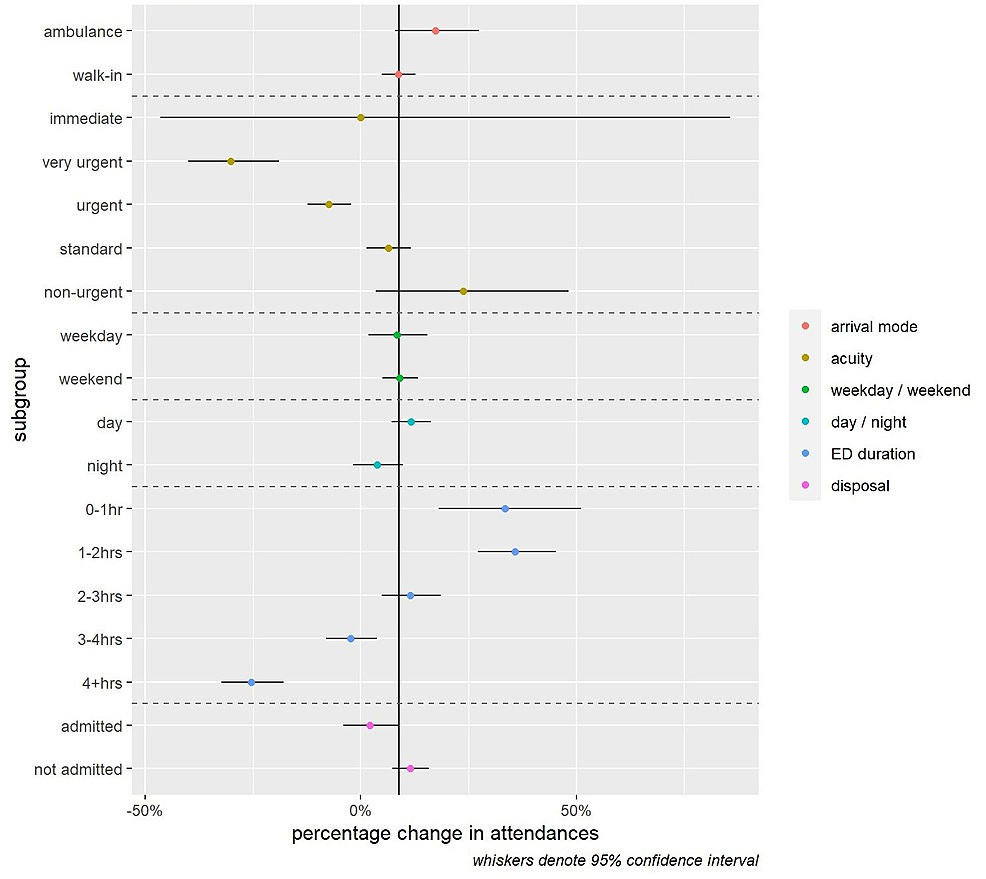
Change in ED attendances for infants with feeding-related problems by patient characteristics: 48 major EDs in England (April 2, 2019 to March 10, 2020) vs (April 1, 2020 to March 10, 2021).

There were substantial differences in the change of infant feeding presentations by three attendance characteristics: acuity, duration, and disposal. Attendances of the following types reduced or increased at a significantly slower rate: very urgent acuity (-30.2%, 95% CI -40.0% to -19.0%), urgent acuity (-7.4%, 95% CI -12.3% to -2.2%), long ED duration (3-4hrs -2.3%, 95% CI -8.1% to 3.8% and 4+hrs -25.4%, 95% CI -32.3% to -17.8%) and those resulting in admission (+2.0%, 95% CI -4.1% to 8.8%). We observed no significant variation in the increase in infant feeding presentations by arrival mode, part of the week (weekday, weekend), or part of the day (8 am-8 pm, 8 pm to 8 am) (Figure 5).

Relationship to numbers of births

Our study reports an increase in the counts of infant feeding-related ED presentations. This leaves open the possibility that this might be driven by an increase in the number of infants. However, we note that the number of live births in 2020 was 4.1% lower than in 2019 and 6.5% lower than in 2018 [21]. Live births increased somewhat in 2021 but remained well below levels in 2019 [22]. The number of low birth weight (<2,500g) and pre-term (<37 weeks) babies was also lower in 2020 than in either of the previous two years. 

Relationship to breastfeeding

**Figure 6:**
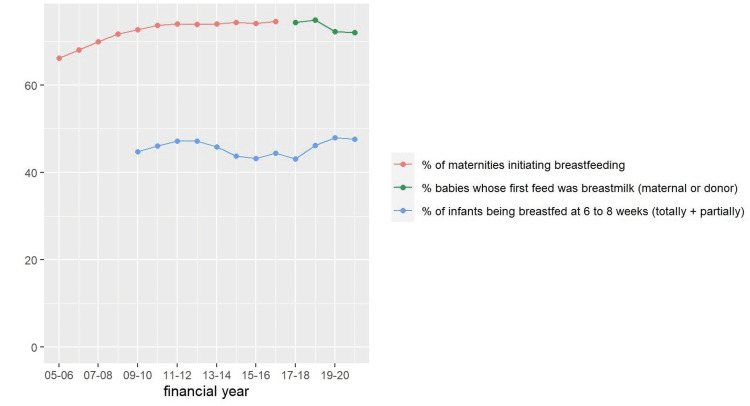
Breastfeeding statistics: England 2005-06 to 2020-21.

Figure 4 shows the percentage of babies whose first feed was breastmilk steadily increased between 2005-06 and 2017-18 to approximately 75% but dropped slightly to 72% in 2019-20 and 2020-21. The percentage of babies still being breastfed (totally or partially) at 6-8 weeks is substantially lower, rising from 41% in 2017-18 to 49% in 2019-21 (Figure 6).

## Discussion

Summary of main findings

Substantial and sustained reductions in ED attendance following the outbreak of SARS-CoV-2 have been widely reported. We find that infant feeding-related ED presentations bucked this trend, increasing by 8.9% in a broadly representative sample of 48 major EDs in the 50-week period following the introduction of COVID-19 lockdown measures in England. These increases were more pronounced amongst first babies, and where the stay in hospital after birth was brief. Our analysis suggests many of these attendances were of low acuity.

Relationship with existing literature

A recent study found that neonatal visits to an ED in a tertiary center in Italy, for minor conditions including feeding issues, increased slightly in March and April 2020 compared to 2019 [23]. Single, multi-site and regional studies from England have also indicated increases in infant presentations to ED during 2020 [6,7,24]. These observations are not isolated to Europe, with a recent Australian single center study showing a substantial increase in infant feeding-related admissions in 2020. Contrary to our data, the Australian study suggests that, amongst other factors, admissions were linked with maternal mental health concerns [25].

All quoted studies to date suggest this increase is likely due to the loss of face-to-face support for families in both formal (e.g., health visitors and primary care) and informal (e.g., community and voluntary sector support groups) settings due to pandemic related service disruptions [6,7,23,26,27].

Possible explanations

We set out below, some possible explanations for our findings. These are guided by a framework that suggest that changes in healthcare activity during the pandemic may be driven by (i) the impact of policy choices, (ii) changes in patient behavior, or (iii) changes in the levels and types of morbidity [28].

During the pandemic, most inpatient intrapartum obstetric and midwifery services continued to receive patients as before, although specific mitigations were introduced to reduce the risk of SARS-CoV-2 transmission [27]. Early discharge from hospital was encouraged [29,30]. The duration of hospital stay following childbirth has decreased steadily over the last few decades to improve NHS maternity services efficiency [31,32]. However, studies have shown that shorter hospital stays following childbirth are associated with an increased risk of readmission for feeding problems and jaundice and a recent single center English study noted the number of neonates attending ED for low acuity issues has steadily increased since 2005 [26,33-35]. In addition, and contrary to national policy and WHO guidelines, there were reports that some hospitals separated newborns from mothers soon after birth, in an attempt to reduce SARS-CoV-2 transmission risks [36-38]. Both shorter than optimal hospital stays, and mother-newborn separations constitute major barriers to establishing breastfeeding.

Ante-natal and post-natal services were substantially disrupted during the pandemic. A survey of healthcare professionals working in maternity units reported reductions in ante-natal and post-natal visits [39]. Health visiting services which provide practical support to parents on issues such as infant feeding were also disrupted. During the first wave of the pandemic, a large number of health visitors were redeployed to respond to the healthcare emergency and as a consequence many aspects of health visiting were suspended or delivered virtually [40]. GP services, a source of holistic care for new families and their babies, changed markedly. One study found face-to-face consultations in a GP practice in England fell by 92.5% in the second half of March 2020 whereas the number of telephone consultations increased by 85.6% [41]. As noted earlier, lockdown measures disrupted many forms of informal and familial support that would normally be available to women who had recently given birth [42]. Parental fear of the consequences of failing to seek appropriate support may also have played a part [43]. 

Remote support services, initiated by voluntary-sector organizations, helped to plug this gap by providing support with infant feeding and specifically breastfeeding following hospital discharge. But access was contingent on both awareness of these support services and availability of information technology and is therefore unlikely to have been uniform and comprehensive. While remote support may have been helpful, the evidence of its effectiveness is limited [44]. 

Trends over the last 15 years show a slight increase in the proportion of babies in England receiving breastmilk at birth. However, this has not been matched by an increase in breastfeeding prevalence rates at 6-8 weeks post-partum, which remain below 50% (Figure 4). These figures reflect the difficulty many women in England have in successfully establishing and sustaining breastfeeding. An in-depth survey in 2010 indicated as many as 80% of women reported they stopped breastfeeding before they wished, citing problems with breastfeeding and lack of support [45]. In 2020-21 the percentage of babies at 6-8 weeks (Figure 4) receiving either partial or total breastmilk was the same as 2019-20 and did not follow the upward trend of previous years. While difficult to prove causation, a plausible reason for this plateau could be the loss of face-to-face support for families due to pandemic-related service reconfiguration. This perspective is supported by the finding that growth in infant feeding ED attendance rates were higher for new parents, than those with older children. Taken together, it appears that reductions in intrapartum length of stay, the loss of informal and formal support services, coupled with historically patchy infant feeding support, led to an increase in the prevalence of infant feeding issues during the pandemic. 

Given the lack of viable alternatives, many of these cases appear to have presented to ED. Unlike many other services, EDs are open to anyone, at any time [46-48]. This was recently referred to as the “ED brand,” trusted by the public “…when all other agencies just don’t or won’t respond…” [49]. Although available, EDs rarely represent the optimal solution for infant feeding issues. Staff may not have the necessary experience or knowledge for effective management, giving rise to increased medicalization of normal childhood behaviors or conditions and further driving health seeking behavior [50]. EDs in England have been under severe pressure for some time, with the proportion of patients waiting more than 4 hours from arrival to discharge reaching record levels in recent months [51]. Increases in infant-feeding presentations will have compounded these challenges.

Policy or practice implications

Our analysis highlights that even before pandemic related changes occurred, many families were attending ED’s across England with infant feeding problems. This suggests a lack of accessible support already existed and which was further compounded by the loss of support mechanisms (face to face care and social interaction) during 2020-21. While health seeking behavior and decision making of families is multi-factorial it is clear that parents took the unenviable decision to attend ED’s for feeding issues, risking SARS-CoV-2 infection.

Our analysis reinforces the need to prioritize infant feeding services when planning responses to future pandemics. We note that the Emergency Nutrition Network issues similar guidance to countries experiencing the effects of natural and man-made disasters [52]. But it should also trigger action to improve the accessibility, coverage and quality of infant feeding services in England in “normal times.” Infant feeding presentations to ED are common. This suggests infant feeding issues are often not identified sufficiently early, and when infant health deteriorates, parents are not able to access a sufficiently responsive specialist service to provide the support and advice required. 

Improving the quality and availability of infant feeding services will also tackle England’s low breastfeeding rates. In 2016, UNICEF encouraged the government to take specific steps to create a supportive, enabling environment for women who want to breastfeed. This includes continuity of care and available support from professional roles including Health Visitors and lactation consultants, and joined-up peer support from community and voluntary sector organizations working alongside statutory services [53].

Limitations of the study

All observational studies are vulnerable to bias. While the recording of ED attendances in our primary dataset is considered to be good, the completeness of diagnoses recording is known to be problematic. We sought to minimize the risk of bias by focusing our analysis on a subset of EDs where diagnosis recording is high and consistent. We enriched this dataset by matching ED data with data about the babies’ birth spells. This yielded useful information about the birth spell duration, mother’s age, gravidity and mental health comorbidities, but matching was far from complete and so inferences relating to these variables should be treated with caution. We found changes in ED duration and disposal, of infant feeding attendances. These changes are likely confounded by changes in ED usage and admission thresholds during the early stages of the pandemic.

In order to estimate confidence intervals for changes in counts between two periods, we assumed that counts of attendances vary in accordance with a Poisson distribution. We note that the assumption of event independence may not hold perfectly in our study. Our subgroup analysis explored differences in count changes by several variables (age group, gender, ethnicity, etc.) in turn. Effects observed in one variable may therefore be an expression of effects driven by another correlated variable. Our study did not incorporate any multivariable analysis.

## Conclusions

While ED attendances reduced dramatically and systematically with the COVID-19 pandemic, presentations for infant feeding issues increased across England. Infant feeding-related presentations at ED are common but strategies to manage the impact of the pandemic led to increases in attendances of this type. Health systems should ensure policies adopt a proactive approach around infant feeding and family support in the near- and long-term future, as well as in any future pandemics. Managing infant-feeding problems in primary and community care settings may reduce the number of presentations to over-stretched EDs that are often not well-placed to address these issues.
